# PARP inhibitor Olaparib increases the sensitization to radiotherapy in FaDu cells

**DOI:** 10.1111/jcmm.14929

**Published:** 2020-01-19

**Authors:** Chuan Liu, Neil Gross, Yanshi Li, Guojun Li, Zhihai Wang, Shixun Zhong, Yuncheng Li, Guohua Hu

**Affiliations:** ^1^ Department of Otorhinolaryngology The First Affiliated Hospital of Chongqing Medical University Chongqing China; ^2^ Department of Head and Neck Surgery The University of Texas MD Anderson Cancer Center Houston TX USA; ^3^ Department of Otorhinolaryngology Union Hospital Tongji Medical College Huazhong University of Science and Technology Wuhan China

**Keywords:** DNA damage repair, hypopharyngeal cancer, olaparib, PARP1, radiosensitization

## Abstract

Radioresistance causes a major problem for improvement of outcomes of patients treated with radiation. Targeting for DNA repair deficient mechanisms is a hallmark of sensitization to resistance. We tested whether Olaparib, a (poly) ADP‐ribose polymerase (PARP) inhibitor, can sensitize the radioresistant FaDu cells to radiotherapy. Radioresistant FaDu cells, called FaDu‐RR cells, were used as the radioresistant hypopharyngeal cancer models. The expression of PARP1 was detected in both FaDu and FaDu‐RR cells. The role of Olaparib in radiosensitization was analysed with several assays including clonogenic cell survival, cell proliferation and cell cycle, and radioresistant xenograft. High expression of PARP1 had a significant effect on enhancing radioresistance in FaDu‐RR cells compared with FaDu cells. After treatment of Olaparib, FaDu‐RR cells showed significantly less and smaller surviving colonies, lower proliferation ability and G2/M arrest than those in the group without treatment. Moreover, Olaparib significantly reduced growth of tumours in FaDu‐RR cell xenografts treated with ionizing radiation. Olaparib can significantly inhibit PARP1 expression and consequently has significant effects on radiosensitization in FaDu‐RR cells. These results indicate that Olaparib may help individualize treatment and improve their outcomes of hypopharyngeal cancer patients treated with radiation.

## 1 INTRODUCTION

Hypopharyngeal cancer accounts for approximately 5%‐15% of all head and neck squamous cell carcinoma.^1^ However, the prognosis of this disease was poor with an overall 5‐year survival rate of only 15%‐45% for late stage patients,^2^ since hypopharyngeal cancer shows a tendency of submucosal spread and is often asymptomatic in the early stage.^3^, ^4^ Surgery, such as laryngopharyngectomy and pharyngeal reconstruction, is still the mainstay treatment option. However, radiotherapy (RT) and chemoradiotherapy (CRT) have changed the treatment trends as organ preservation approaches are being increasingly utilized more recently.^5^ A meta‐analysis with 286 patients of hypopharyngeal cancer has shown that chemoradiotherapy may offer similar survivorship compared with surgery in advanced disease, thus making larynx preservation feasible.^6^ Furthermore, salvage radiotherapy had a significant positive impact on 3‐year local progression‐free survival and overall survival for hypopharyngeal squamous cell carcinoma.^7^ Although RT has become a valuable therapeutic option for improving the outcome, the 5‐year survival rate for patients who received radiotherapy without surgery remained only 22.6%.^4^ One of the reasons for unsatisfactory outcome of this treatment is due to radioresistance of tumours. Studies have demonstrated multiple factors, such as hypoxia 8 and EGFR/β,^9^ contribute to the radioresistance of hypopharyngeal cancer, while the mechanisms underlying the radioresistance still remain unclear. Therefore, it is crucial to further investigate the mechanisms underlying the radioresistance of hypopharyngeal cancer treated with radiation.

Ionizing radiation (IR) has genotoxic effects on cancer cells by directly damaging the molecular structure of DNA and consequently inhibits cell proliferation and induces cell death.^10^ Poly (ADP‐ribose) polymerase 1(PARP1) is an ADP‐ribosylating enzyme for initiating various forms of DNA repair, such as base excision repair (BER) and single‐strand break repair.^11^, ^12^ Olaparib, a highly selective potent PARP inhibitor, has shown radiosensitization in multiple cancers, especially in those with BRCA1/BRCA2 mutations.^13^, ^14^, ^15^ While, whether Olaparib can increase the radiosensitivity of hypopharyngeal cancer remains unclear. In this study, we aimed to detect the role of PARP1 in radioresistance and evaluate effect of its inhibitor, Olaparib, on radiosensitization of hypopharyngeal cancer. We have been suggested that high expression of PARP1 enhances the radioresistance and Olaparib has a positive effect on the radiosensitization in hypopharyngeal cancer, providing an effective therapeutic strategy and improving their prognosis of hypopharyngeal cancer patients treated with radiation.

## 2 MATERIALS AND METHODS

### 2.1 Cell culture

FaDu cell lines were obtained from the Cell Bank of Chinese Academy of Sciences. FaDu‐RR cell lines were generated from the parental FaDu cells through the repeated exposure to ionizing radiation with a total dose of 10 Gy (16 MV X‐rays) using a linear accelerator (UNIQUE; Varian) at a dose rate of approximately 3 Gy/min each time, as shown in our previous study.^16^ Both FaDu and FaDu‐RR cells were cultured in DMEM medium (HyClon) with 10% foetal bovine serum (PAN), 100 U/mL penicillin and 100 µg/mL streptomycin, at 37°C in a humidified incubator with 5% CO_2_.

### 2.2 qRT‐PCR

The total RNA was extracted and reversely transcribed using One‐Step SYBR PrimeScript RT‐PCR Kit II (Takara Biotechnology). The qRT‐PCR reaction was performed using SYBR Premix Ex Taq (Takara Biotechnology) according to the manufacturer's instructions. The GAPDH was used as the internal reference as control. The primers were as following: for PARP1 (forward 5′‐CCGCATACTCCATCCTCAGT‐3′ and reverse 5′‐GCTTCTTCATCCCAAAGTCG‐3′), and for GAPDH (forward 5′‐ACCTGACC TGCCGTCTAGAA‐3′ and reverse 5′‐TCCACCACCCTGTTGCTGTA‐3′). The ΔΔCt was used as quantitative threshold for gene expression analysed.

### 2.3 Western blotting

Proteins were extracted from the cells using RIPA buffer (Beyotime Institute of Biotechnology), then were separated using SDS polyacrylamide gel electrophoresis with 30 µg total extract, and finally were transferred to polyvinylidene fluoride (Millipore). After blocked with 5% nonfat‐dried milk, the membranes were incubated with PARP1 (1:100, Santa Cruz Biotechnology) for overnight at 4°C. The β‐actin (1:1000, Biodragon) was used as the internal control. The antibody/antigen complexes were revealed by the ECL (Advansta). Membranes were scanned and analysed using ChemiDoc Touch Imaging System (Bio‐Rad) with the Image Lab software.

### 2.4 Immunofluorescence

The cells were seeded on a covered glass and then divided into the three groups. Two groups were treated with ionizing radiation with a dose of 10 Gy. Then, the cells with non‐irradiation, 30 minutes after irradiation and 12 hours after irradiations were fixed with 4% paraformaldehyde for 30 minutes and permeabilized with 0.5% Triton X‐100 solution for 1 hour. The cells were incubated with PARP1 (1:30, Santa Cruz Biotechnology) for overnight at 4°C, and then secondary antibodies (1:1000, Alexa Fluor 568, Life Technologies). Counterstaining with DAPI and neutral resins mount was performed. Images of cells were captured by the Leica DM4B microscope (Leica Microsystems).

### 2.5 Clonogenic cell survival assay

FaDu‐RR cells were seeded at 1000, 2000, 4000, 6000, 8000 and 10 000 cells/well at 6‐well plate, respectively. The cells in the experiment group were treated with 2 μg/mL Olaparib (MedChemExpress) for 24 hours. Then, FaDu‐RR cells with and without treatment of Olaparib were irradiated with 16 MV X‐rays using a linear accelerator (UNIQUE; Varian) with a dose of 3 Gy/min at the dose corresponding to 0, 1, 2, 4, 6, 8 and 10 Gy, respectively. The cells were cultured in a humidified incubator with 5% CO_2_ at 37°C for 14 days, and then the cells were fixed with 75% ethanol and haematoxylin.

### 2.6 Cell proliferation assay

Both FaDu‐RR cells and FaDu‐RR cells treated with Olaparib for 24 hours were seeded into 96‐well plates in a density of 2000 cells/well, and cultured at 37°C with DMEM containing 10% DMSO as a control. After irradiated with 10 Gy X‐ray (0 day, 1 day, 2 days, 3 days, 4 days, 5 days), 10 μL of CCK‐8 was added into each well and incubated with 5% CO_2_ at 37°C for 1 hour. The absorbance at 450 nm was measured using a 96‐well microplate reader. Survival rate was calculated as follows: (OD values of the experimental samples/OD values of the control) × 100%.

### 2.7 Cell cycle assay

The FaDu‐RR cells were seeded into 6‐well plate, and one group of FaDu cells were treated with Olaparib (2 μg/mL) for 24 hours. The cells with and without treatment of Olaparib were ionizing radiated with a dose of 10 Gy and incubated at 37°C. The cells without irradiation and 30 minutes after irradiation were then digested with trypsin and fixed with ice‐cold 70% ethanol, incubated with propidium iodide (PI) solution (Invitrogen) and analysed by FACSVantage SE system (BD Biosciences). All above experiments were replicated in triplicate.

### 2.8 Xenograft studies

All animal experiments were performed under a protocol approved by the Institutional Animal Care Committee at Chongqing Medical University. A group of 5‐week, male, athymic nude mice were received 5 × 10^6^ FaDu‐RR cells on the left flank by subcutaneous injections. The eight treated mice then were randomized into two groups as the treated group with Olaparib and non‐treated group without Olaparib. The group treated with Olaparib were received intraperitoneal injection of Olaparib (10 μg/g) per time at days 14, 18, 21 and 25, respectively. Both two groups received a dose of 2 Gy of radiation per time at days 15, 19, 22 and 26, respectively. Tumours were measured once a week using a digital caliper. Tumour volume was calculated as: 1/2 × length × width^2^. The mice were sacrificed, and tumours were harvested at days 26 after treatment. Tumour samples were then collected and immediately frozen in liquid nitrogen.

### 2.9 Statistical analysis

All data were presented as the mean ± standard deviation (SD) and were analysed with statistical software SPSS 21.0 (SPSS Inc). Comparisons of continuous variables between the groups were performed using the Student's *t* test. The criterion for statistical significance was taken at *P* < .05.

## 3 RESULTS

### 3.1 Overexpression of PARP1 in FaDu‐RR cells

**Figure 1:**
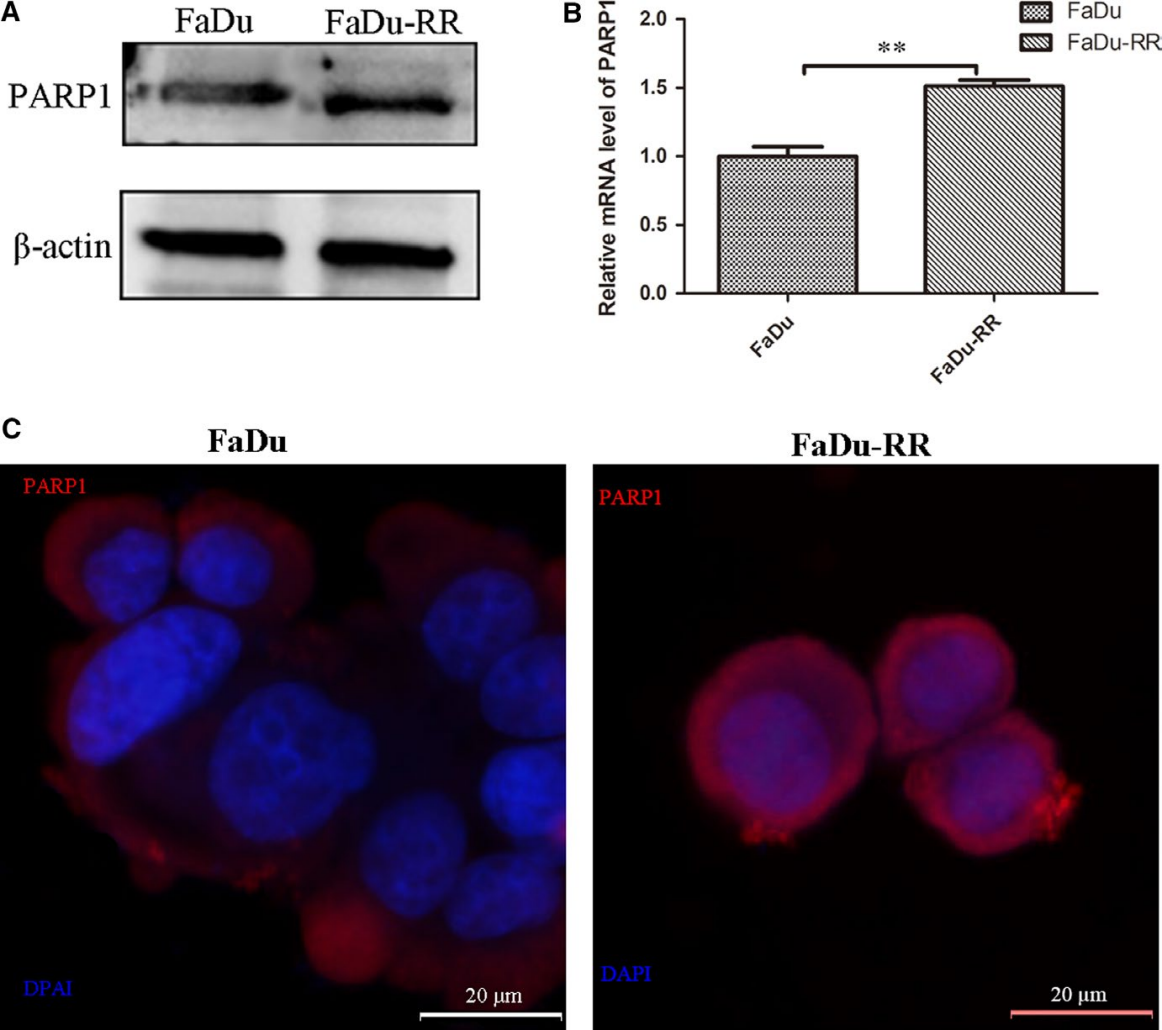
Demonstration of high expression of PARP1 in radioresistant FaDu‐RR cells by Western blot (A), qRT‐PCR (B) and immunofluorescence (C). **P < .01

As shown in Figure 1A,C, the protein levels of PARP1 were increased in the FaDu‐RR cells compared with those in the FaDu cells. The mRNA level of PARP1 was also significantly higher in the FaDu‐RR cells than that in FaDu cells (Figure 1B). These results indicated that high expression of PARP1 had a positive effect on radioresistance in the FaDu cells.

### 3.2 Inhibition of Olaparib in PARP1 expression in FaDu‐RR cells

**Figure 2:**
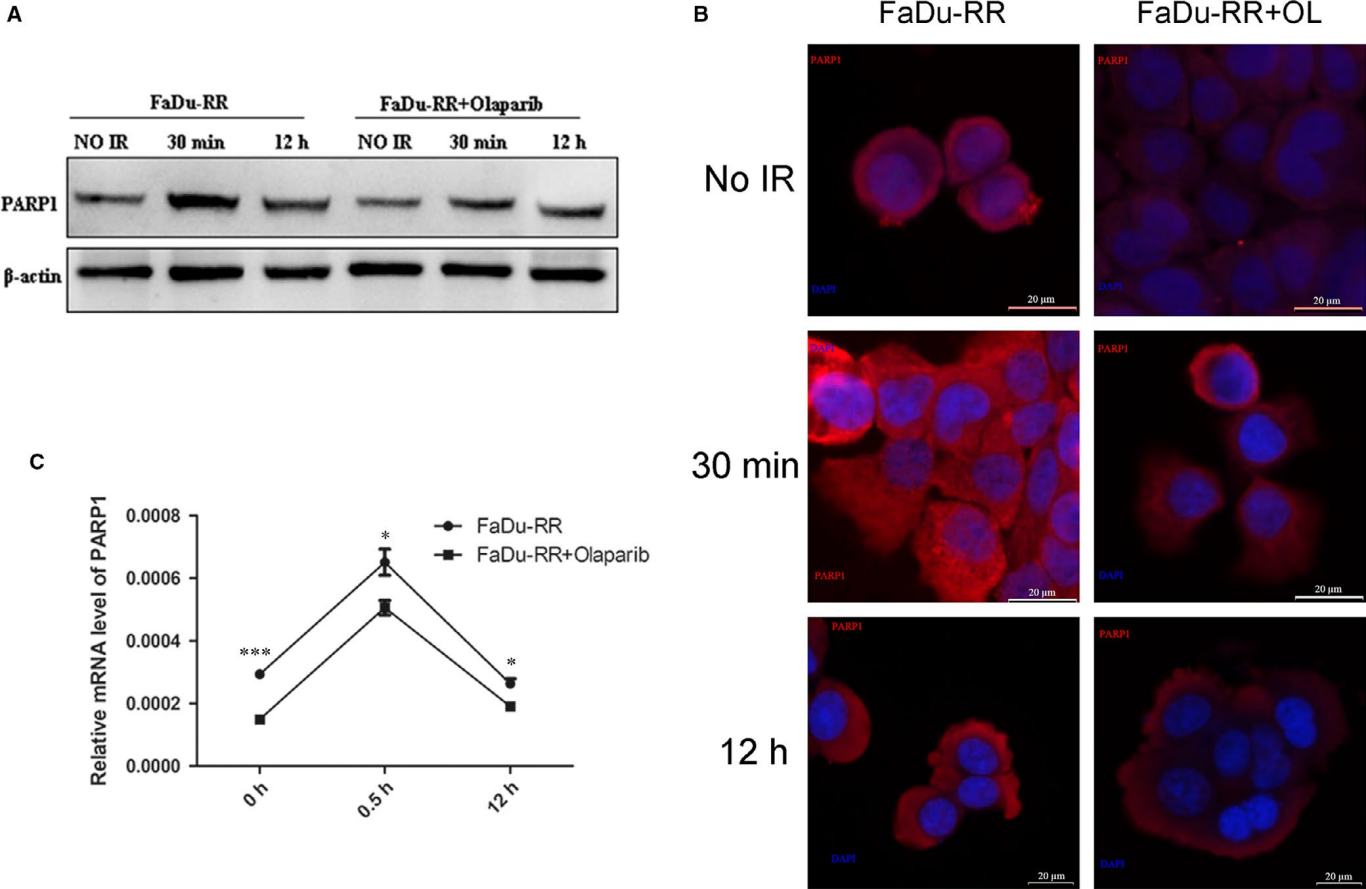
Inhibition of Olaparib in expression of PARP1 with IR at 0 min,, 30 min, and 12 h, respectively by Western blot (A), qRT‐PCR (B), and immunofluorescence (C). *P < .05 and ***P < .001

As shown in Figure 2A,C, the protein expression of PARP1 was decreased in the Olaparib‐treated group without IR, while the expression of PARP1 significantly increased in both groups in 30 minutes after IR. Moreover, the expression was significantly higher in non‐treated group than that in Olaparib‐treated group. At 12 hours after irradiation, the expression of PARP1 decreased in both groups, but remained higher in non‐treated group. The mRNA expression level of PARP1 showed the same trend (Figure 2B). These results indicated that Olaparib could effectively inhibit the level of PARP1 in FaDu‐RR cells both before and after irradiation.

### 3.3 Increased radiosensitivity of FaDu‐RR cells by olaparib

**Figure 3:**
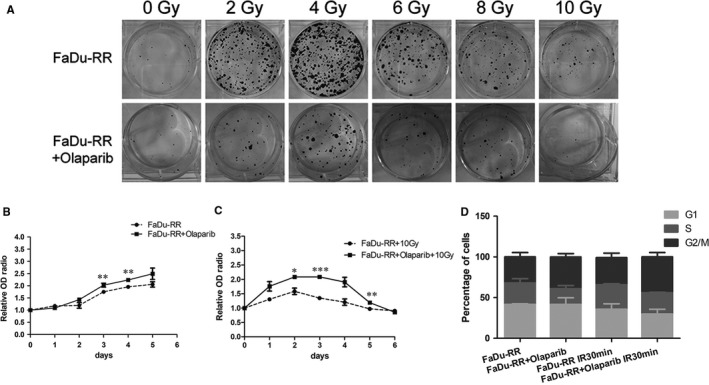
Enhanced radiosensitivity of Olaparib in FaDu‐RR cells. In clonogenic cell survival assay, the Olaparib‐treated group had less and smaller surviving colonies (A). In cell proliferation assay, the Olaparib‐treated group showed similar proliferation ability before IR (B) but significantly lower proliferation ability after IR (C). In cell cycle analysis, Olaparib‐treated group showed significant G2/M arrest both before and after IR (D)

As shown in Figure 3A, the surviving colonies had no significant difference between FaDu‐RR cells with and without treatment of Olaparib before irradiation. However, the surviving colonies were much more and bigger in FaDu‐RR cells than those in FaDu‐RR cells treated with Olaparib after irradiation, indicating the radiosensitivity effect of Olaparib. For the cell proliferation as shown in Figure 3B, there was no significant difference between FaDu‐RR cells treated with and without Olaparib at the first 2 days, while at the third day, the proliferation ability of FaDu‐RR cells treated with Olaparib was higher than that in non‐treated FaDu‐RR cells. After irradiated with a dose of 10 Gy X‐ray (Figure 3C), the proliferation ability of both groups of FaDu‐RR cells significantly decreased. However, FaDu‐RR cells treated with Olaparib decreased more sharply in the later days, especially at day 6 after irradiation, showing Olaparib‐treated group was more sensitive to radiotherapy. All these results supported the role of Olaparib in increase of radiosensitivity in FaDu‐RR cells.

### 3.4 G2/M phase arrest in FaDu‐RR cells by olaparib

As shown in Figure 3D, the FaDu‐RR cells treated with Olaparib revealed a significant decrease in S phase, which was within the radioresistant phases of cell cycle, but increase in G2/M phase, which was within the radiosensitive phases of cell cycle. After irradiated with a dose of 10 Gy X‐ray, both FaDu cells treated with and without Olaparib showed a decrease in G1 phase but an increase in both S phase and G2/M phase. However, the percentage of S phase was significantly lower in FaDu‐RR cells treated with Olaparib than that in non‐treated FaDu‐RR cells, demonstrating FaDu‐RR cells treated with Olaparib were much more sensitive to IR.

### 3.5 Enhanced radiosensitivity of FaDu‐RR cells in xenograft by olaparib

**Figure 4:**
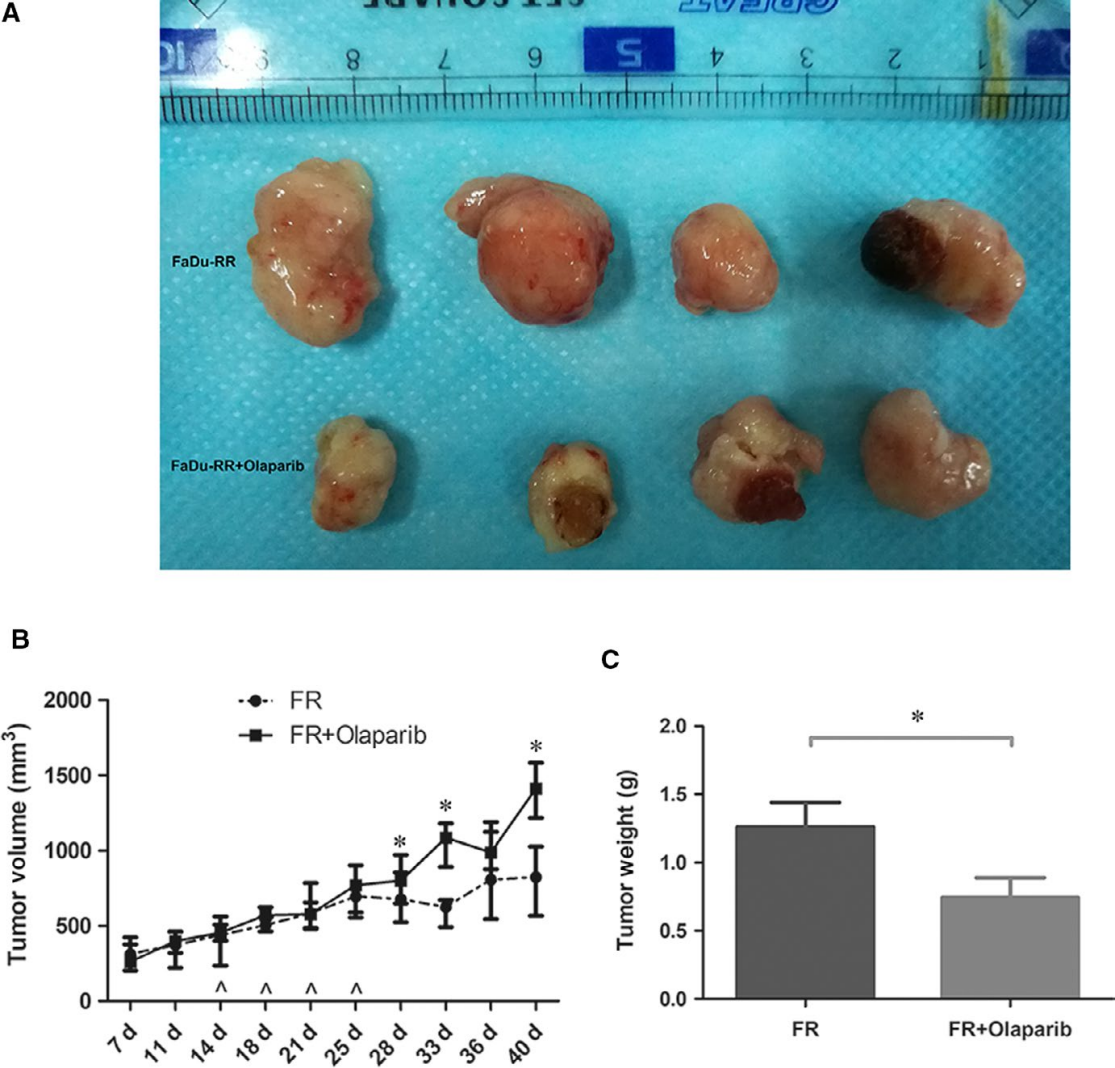
Increased radiosensitivity of FaDu‐RR cells in xenografts by Olaparib. (A) Tumour sizes in Olaparib‐treated group were significant smaller. (B) The tumour growth rate was slower in Olaparib‐treated group. (C) Tumour weights in Olaparib‐treated group were significant lighter. *P < .05

As shown in Figure 4A,C, treatment with Olaparib resulted in significantly smaller tumours in the xenografts injected with FaDu‐RR cells. Such a treatment also significantly reduced the growth of FaDu‐RR cells with irradiation in the xenografts (Figure 4B). These results indicated that treatment of Olaparib can increase the radiosensitivity of FaDu‐RR cells in vivo.

## 4 DISCUSSION

Radiosensitivity is typically affected by various factors including tumour intrinsic radiosensitivity, re‐oxygenation process, cell cycle redistribution, tumour tissue repopulation and tumour repair capacity.^17^ Ionizing radiation induces several types of DNA damage, including base modifications, crosslinks, single‐strand breaks (SSB) and double‐strand breaks (DSB).^18^ The failure of DNA damage response (DDR) can trigger a permanent cell cycle arrest or programmed cell death.^19^ Increased DNA repair capacity is a hallmark of radiotherapy resistance. Thus, there is substantial clinical need for potent and effective biomarkers for individual response to radiotherapy.

PARP1, belonged to the marvellous PARPs family, is an abundant nuclear protein which binds to both DNA SSB and DSB through its *N*‐terminal zinc fingers.^20^ The changes by binding increase its catalytic activity at the *C*‐terminal to hydrolyse NAD^+^ and produce liner and branched polymers of ADP‐ribose (PAR) chains that can extend over hundreds of ADP‐ribose units.^21^, ^22^, ^23^ Studies have shown that PARP1 initiates and modulates multiple DNA repair pathways. PARP1 may recruit BER complexes for base modification.^11^ PARP1 ADP‐ribosylation activity could be enhanced by interaction with Ku, forming a functional complex with DNA‐dependent protein kinase (DNA‐PK) and participating in nonhomologous end joining (NHEJ).^24^ PARP1 may also bind to short single‐stranded overhangs and recruit Mre11 in the homologous recombination (HR).^25^ Thus, PARP1 plays a crucial role in maintaining genomic integrity. There was evidence that the absence of PARP1 produced hypersensitivity to ionizing radiation.^26^ In the current study, overexpression of PARP1 was detected in radioresistant FaDu cells, demonstrating a more active DNA repair ability in radioresistant FaDu cells. Therefore, we speculated the inhibition of PARP1 can help sensitize FaDu cells to radiation.

Olaparib as the most representative of PARP1 inhibitors interferes with BER pathway and subsequently leads to the accumulation of unrepaired SSBs, which provoke collapsed replication forks in S phase, formatting the deleterious DSB.^27^ In the cells with defective HR, the DSB resulted from Olaparib can be either repaired by more error‐prone DNA repair mechanisms such as NHEJ or remain unrepaired, leading to synthetic lethality.^28^, ^29^ It has been approved as an anticancer drug for treatment in ovarian and BRCA mutated cancers. Moreover, radiosensitization enhanced by Olaparib has also been observed in several types of tumours. In the current study, we found Olaparib significantly inhibited PARP1 expression and consequently showed a strong radiosensitive effect on FaDu‐RR cell and the radioresistant xenograft. In medulloblastoma, ependymoma and high grade glioma cell lines, sensitization to radiation was shown with sub‐cytotoxic concentrations of Olaparib.^30^ In HR‐deficient head and neck cancers, Olaparib enhanced the radiotherapeutic ratio via disabling DNA replication processes.^31^ Moreover, recent studies have been focused on the combination of Olaparib with chemotherapeutic agents in cancer treatment.^32^, ^33^ These combinations offer the prospect to broaden the clinical benefit of Olaparib beyond its use as monotherapy. Although we have demonstrated the radiosensitization by Olaparib in radioresistant FaDu cells, the comprehensive mechanism remains a challengeable topic for future research. The further studies, such as dose‐time effect, pre‐clinical analysis, are still needed. Moreover, our current finding also should be validated in other hypopharyngeal cancer cell lines once they have become available.

In conclusion. Taken together, our study demonstrated high expression of PARP1 in radioresistant FaDu‐RR cells could serve as a potential radiosensitivity biomarker for hypopharyngeal carcinoma. Our findings from the current study support that Olaparib can significantly inhibit PARP1 expression and consequently sensitize FaDu‐RR cell to radiotherapy, which may help individualize treatment for improved outcomes of patients with hypopharyngeal carcinoma treated with radiotherapy. These findings may also lay the foundation for future studies on assessment in utilization of Olaparib in patients with hypopharyngeal carcinoma.

## CONFLICT OF INTEREST

The authors confirm that there are no conflicts of interest.

## AUTHORS' CONTRIBUTIONS

CL performed the cell experiments and drafted the article. NG and GJL revised the work. YSL and WZH performed the xenograft experiments. SXZ and YCL performed the analysis. GHH performed the interpretation of data. All authors read and approved the final manuscript.

## Data Availability

The data that support the findings of this study are available on request from the corresponding author. The data are not publicly available due to privacy or ethical restrictions.
